# Pre-existing Health Conditions and Epicardial Adipose Tissue Volume: Potential Risk Factors for Myocardial Injury in COVID-19 Patients

**DOI:** 10.3389/fcvm.2020.585220

**Published:** 2021-01-11

**Authors:** Zhi-Yao Wei, Rui Qiao, Jian Chen, Ji Huang, Wen-Jun Wang, Hua Yu, Jing Xu, Hui Wu, Chao Wang, Chong-Huai Gu, Hong-Jiang Li, Mi Li, Cong Liu, Jun Yang, Hua-Ming Ding, Min-Jie Lu, Wei-Hua Yin, Yang Wang, Kun-Wei Li, Heng-Feng Shi, Hai-Yan Qian, Wei-Xian Yang, Yong-Jian Geng

**Affiliations:** ^1^State Key Laboratory of Cardiovascular Disease, Department of Cardiology, Center for Coronary Heart Disease, National Center for Cardiovascular Diseases of China, Fu Wai Hospital, Peking Union Medical College, Chinese Academy of Medical Sciences, Beijing, China; ^2^Department of Cardiology, Anqing Hospital, Anhui Medical University, Anqing, China; ^3^Guangdong Provincial Key Laboratory of Biomedical Imaging, Department of Cardiovascular Medicine, Fifth Affiliated Hospital of Sun Yat-sen University, Zhuhai, China; ^4^Department of Cardiology, Beijing Anzhen Hospital, Capital Medical University, Beijing, China; ^5^Department of Cardiology, Daye Chinese Medicine Hospital, Daye, China; ^6^Division of Life Sciences and Medicine, Department of Cardiology, The First Affiliated Hospital of University of Science and Technology of China, Hefei, China; ^7^Division of Life Sciences and Medicine, Department of Infectious Diseases, The First Affiliated Hospital of University of Science and Technology of China, Hefei, China; ^8^Institute of Cardiovascular Disease, China Three Gorges University, Yichang, China; ^9^Department of Cardiology, Yichang Central People's Hospital, Yichang, China; ^10^Coronary Care Unit, Baoding No.1 Central Hospital, Baoding, China; ^11^Sixth Department of Hepatopathy, Baoding People's Hospital, Baoding, China; ^12^Department of Gastroenterology, Yingcheng Chinese Medicine Hospital, Yingcheng, China; ^13^Department of Otolaryngology, Daye People's Hospital, Daye, China; ^14^Department of Radiology, Infection Hospital of Anhui Provincial Hospital (Hefei Infectious Diseases Hospital), Hefei, China; ^15^State Key Laboratory of Cardiovascular Disease, Department of Magnetic Resonance Imaging, National Center for Cardiovascular Diseases of China, Fu Wai Hospital, Peking Union Medical College, Chinese Academy of Medical Sciences, Beijing, China; ^16^State Key Laboratory of Cardiovascular Disease, Department of Radiology, National Center for Cardiovascular Diseases of China, Fu Wai Hospital, Peking Union Medical College, Chinese Academy of Medical Sciences, Beijing, China; ^17^State Key Laboratory of Cardiovascular Disease, Medical Research and Biometrics Center, National Center for Cardiovascular Diseases of China, Fu Wai Hospital, Peking Union Medical College, Chinese Academy of Medical Sciences, Beijing, China; ^18^Department of Radiology, Fifth Affiliated Hospital of Sun Yat-sen University, Zhuhai, China; ^19^Department of Radiology, Anqing Hospital, Anhui Medical University, Anqing, China; ^20^Division of Cardiology, Department of Internal Medicine, The Center for Cardiovascular Biology and Atherosclerosis Research, McGovern Medical School, University of Texas Health Science Center at Houston, Houston, TX, United States

**Keywords:** COVID-19, SARS-CoV-2, pandemic (COVID-19), CT imaging findings, cardiac complication

## Abstract

**Background:** Myocardial injury is a life-threatening complication of coronavirus disease 2019 (COVID-19). Pre-existing health conditions and early morphological alterations may precipitate cardiac injury and dysfunction after contracting the virus. The current study aimed at assessing potential risk factors for COVID-19 cardiac complications in patients with pre-existing conditions and imaging predictors.

**Methods and Results:** The multi-center, retrospective cohort study consecutively enrolled 400 patients with lab-confirmed COVID-19 in six Chinese hospitals remote to the Wuhan epicenter. Patients were diagnosed with or without the complication of myocardial injury by history and cardiac biomarker Troponin I/T (TnI/T) elevation above the 99th percentile upper reference limit. The majority of COVID-19 patients with myocardial injury exhibited pre-existing health conditions, such as hypertension, diabetes, hypercholesterolemia, and coronary disease. They had increased levels of the inflammatory cytokine interleukin-6 and more in-hospital adverse events (admission to an intensive care unit, invasive mechanical ventilation, or death). Chest CT scan on admission demonstrated that COVID-19 patients with myocardial injury had higher epicardial adipose tissue volume ([EATV] 139.1 (83.8–195.9) vs. 92.6 (76.2–134.4) cm^2^; *P* = 0.036). The optimal EATV cut-off value (137.1 cm^2^) served as a useful factor for assessing myocardial injury, which yielded sensitivity and specificity of 55.0% (95%CI, 32.0–76.2%) and 77.4% (95%CI, 71.6–82.3%) in adverse cardiac events, respectively. Multivariate logistic regression analysis showed that EATV over 137.1 cm^2^ was a strong independent predictor for myocardial injury in patients with COVID-19 [OR 3.058, (95%CI, 1.032–9.063); *P* = 0.044].

**Conclusions:** Augmented EATV on admission chest CT scan, together with the pre-existing health conditions (hypertension, diabetes, and hyperlipidemia) and inflammatory cytokine production, is associated with increased myocardial injury and mortality in COVID-19 patients. Assessment of pre-existing conditions and chest CT scan EATV on admission may provide a threshold point potentially useful for predicting cardiovascular complications of COVID-19.

## Introduction

Coronavirus disease 2019 (COVID-19) is a highly contagious disease caused by severe acute respiratory syndrome coronavirus 2 (SARS-CoV-2). Since its first breakout in Wuhan, China, the COVID-19 pandemic has triggered a worldwide health crisis. According to WHO, globally, as of September 20, 2020, COVID-19 has caused nearly one million deaths (1). SARS-CoV-2 mainly attacks the respiratory system, clinically characterized by rapidly progressive pneumonia and acute respiratory distress syndrome (ARDS) (2). However, the virus may damage other tissues and organs directly or indirectly, in particular, the cardiovascular system. Indeed, individuals with pre-existing health conditions are highly vulnerable to the pathological insults from the viral infection (3, 4). COVID-19 patients display not only the manifestations of pulmonary injury but also multiple organ damage and dysfunction. The viral injury to various tissue or organs constitutes a complex clinical syndrome with a broad spectrum of pathophysiological characteristics, which contribute to the severity and mortality of COVID-19 (5–8).

Currently, COVID-19 patients with myocardial injury are diagnosed when the serum levels of troponin I/T (TnI/T) increase above the 99th percentile upper reference limit, after excluding TnI/T elevation and other evidence related to pre-existing obstructive coronary artery disease. Thus, the abnormal levels of myocardial biomarkers constitute the main criteria to identify COVID-19 patients with myocardial injury. However, TnI/T changes may occur in other pathological conditions, such as infection, hypoxia, and renal insufficiency, commonly observed during the development of COVID-19. Hence, assessment of myocardial injury should be performed using a comprehensive approach, including non-invasive imaging, electrocardiography, and laboratory examination for proper clinical judgment in patients with abnormal TnI/T levels. Regarding cardiac morphological examination or image analysis, echocardiography or cardiovascular magnetic resonance (CMR) is not routine examination for COVID-19 patients, and generates non-specific images that may be lagging in early detection of myocardial injury (9). Conversely, chest computed tomography (CT) is routinely performed in patients suspected for COVID-19, usually as soon as hospital admission, to evaluate the severity of pneumonia. Therefore, an early imaging indicator based on chest CT is valuable for timely assessment and diagnosis of myocardial injury morphologically. Epicardial adipose tissue volume (EATV) has been used to evaluate the adipose tissue between the epicardial surface and pericardium, and reportedly associated with heart inflammation (10). In this multi-center, retrospective study, we explored the pre-existing health conditions and chest CT EATV as potential risk factors for myocardial injury in COVID-19 patients.

## Methods

### Study Design, Participants, and Data Recording

The current multi-centered, retrospective study of laboratory-confirmed COVID-19 patients was conducted in six independent hospitals, located in the Eastern, Southern, Northern, and Central regions of China. All the cases of COVID-19 were confirmed positively in SARS-CoV-2 detection of respiratory specimens by real-time reverse-transcriptase–polymerase-chain-reaction (RT-PCR), according to the guidelines of the World Health Organization and the National Health Commission of China (11, 12). A total of 549 consecutive patients with confirmed COVID-19 were admitted from January 3 to February 26, 2020. Except for 43 patients who remain hospitalized and 106 patients with no record of TnI/T, all other 400 patients were enrolled in the final analysis.

The epidemiological, demographic, clinical, laboratory, imaging, treatment, and outcome data of enrolled patients were collected by experienced local clinicians, and entered into a computerized database and cross-checked. The time from the onset of symptoms to hospital admission was 5 (3–7) days. All the patients underwent at least one TnI/T test, 285/400 (96.3%) patients had TnI/T data available within the first 24 h of hospital admission, and 373/400 (93.3%) patients had more than one test result of TnI/T during hospitalization. Myocardial injury was diagnosed and confirmed according to the highest level of TnI/T during hospitalization.

### Study Definitions

Myocardial injury was diagnosed when the highest level of Troponin I/T (TnI/T) was above the 99th percentile upper reference limit (reference range of each hospital is available at Supplementary Table 1), after excluding the possibility of acute coronary syndrome (13). Fever was defined as an axillary temperature of 37.3°C or higher. Hypertension was defined as systolic blood pressure over 140 mmHg or diastolic blood pressure over 90 mmHg. In-hospital adverse events included admission to an intensive care unit (ICU), the use of invasive mechanical ventilation, or death (14, 15). The injury was further confirmed by reviewing admission logs and histories from electronic medical care records.

### Analysis of Epicardial Adipose Tissue Volume (EATV) by CT Scan

Chest CT scan was performed within the first 24 h of hospital admission in accordance with the guidance for COVID-19 from the Chinese National Health Commission (12). Chest CT images were collected, and measured using breath-hold electrocardiogram-gated CT scanners with 256 or 64 detector rows (uCT 760, uMI 780 scanners, United Imaging, Shanghai, China; Precision 32, CAMPO Imaging, Shenyang, China; NeuViz 64 In/En, Neusoft, Liaoning, China; SOMATOM Emotion 16, Siemens, Germany; SOMATOM definition AS, Siemens, Germany; Optima CT680, GE Healthcare, USA). The scan conditions were set as 120–140 kV, 300–320 mA, 512 × 512 matrix, and the field of view was 240 mm with a slice thickness of 1–3 mm. Images were reconstructed using a soft-tissue algorithm. EATV was calculated and established from mediastinal window images according to the standardized operation protocol by trained radiologists blinded to the study protocol. The baseline characteristics of patients with and without EATV were roughly the same (Table 1). Epicardial adipose tissue was identified on the CT scan as a hypodense rim surrounding the myocardium and limited to the pericardium. The visceral pericardium was traced manually from the aortic arch to the left ventricular apex, and all extra-pericardial tissue was excluded. The individual EATV measurement within the manually traced epicardium in each slice was detected by assigning a threshold CT value of −200 and −30 HU and then was automatically summed with the software of Siemens Syngo.via (Siemens, Germany) to determine the total EATV.

**Table 1:** Baseline comparison between general population (*n* = 400) and patients with EATV value (*n* = 272).

	**Patients with EATV value**	**Patients without EATV value**	***P*-value^**a**^**
Age (yrs)	48.7 ± 15.4	48.0 ± 16.6	0.666
Female	137/272 (50.4%)	54/128 (42.2%)	0.127
Hypertension	45/272 (16.5%)	18/128 (14.1%)	0.525
Diabetes	26/272 (9.6%)	11/128 (8.6%)	0.756
White blood cells, × 10^9^/L	5.0 (4.0–5.7)	5.0 (4.0–6.4)	0.553
Platelets, × 10^9^/L (*n* = 388)	176.0 ± 60.6	167.7 ± 56.0	0.209
Alanine aminotransferase, U/L (*n* = 392)	24.0 (15.0–38.0)	23.0 (15.0–41.0)	0.922
Creatinine, μmol/L (*n* = 391)	54.9 (46.0–67.1)	64.0 (55.0–79.0)	<0.001
C-reactive protein, mg/L (*n* = 393)	17.0 (4.1–69.0)	23.0 (5.7–49.5)	0.082

a *Significant difference (p < 0.05) was determined between patients with EATV value and patients without EATV value*.

### Statistical Analyses

Data were presented as mean ± standard deviation (*SD*) or median with quartiles for continuous variables and number (%) for categorical variables. Differences between patients with and without myocardial injury were assessed with the two-tail *t*-test or Wilcoxon rank-sum test for continuous variables and Chi-square or Fisher's exact test for categorical variables. The receiver operating characteristic (ROC) curve analysis was used to select a cut-off value for EATV, and sensitivity and specificity for predicting myocardial injury incidence were calculated. Multivariate logistic regression analysis was applied to control confounding factors that might be associated with EATV (age, weight, history of hyperlipidemia, and coronary heart disease) when identifying the predicting value of EATV for the incidence of myocardial injury. Multivariate logistic regression analysis was also applied to control baseline confounders (age, history of hypertension, diabetes, and coronary heart disease) when exploring the association of myocardial injury with severe COVID-19. The consistency of the results was confirmed in patients with EATV value in subgroup analysis. Tests were two-sided with significance set at α < 0.05. SPSS for Windows (Version 22.0, IBM) and Graphpad Prism 8.0 software were used for statistical analysis.

## Results

### Baseline Characteristics and Pre-existing Health Conditions

**Figure 1:**
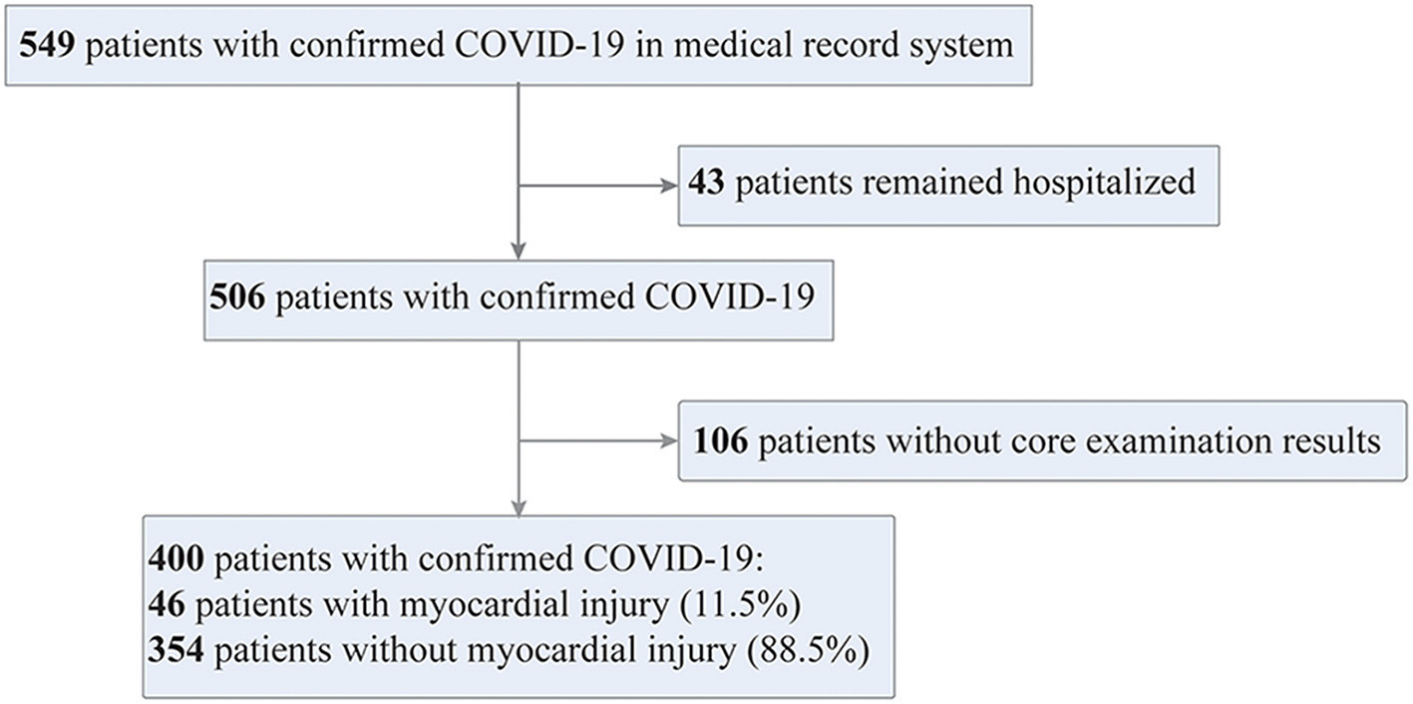
Flowchart of patient recruitment. COVID-19 cases confirmed by RT-PCR assays were enrolled in this cohort study from six hospitals or regional medical centers remote to the Wuhan epicenter from January 3 to February 26, 2020.

The current cohort study enrolled 549 patients consecutively who suffered from laboratory-confirmed COVID-19 and admitted to six hospitals outside of the Wuhan epicenter as of March 8, 2020. Among them, there were 43 patients remained hospitalized and 106 patients with no record of TnI/T and thereby excluded from the study. All other 400 patients were entering into the final analysis, and the enrolling process was shown in Figure 1. There were 46 hospitalized COVID-19 patients were diagnosed suffering from myocardial injury. COVID-19 patients with myocardial injury were slightly older than those without [52.5 (42.8–68.0) vs. 49.0 (36.0–60.0) years]. The incidence of myocardial injury was much higher in patients with pre-existing health conditions, such as hypertension [12/46 (26.1%) vs. 50/354 (14.1%); *P* = 0.035], hyperlipidemia [4/46 (8.7%) vs. 7/354 (2.0%); *P* = 0.028], and chronic kidney disease [3/46 (6.5%) vs. 2/354 (0.6%); *P* = 0.012] as compared with non-myocardial injury COVID-19 patients.

There were no differences in the percentage of patients having the signs and symptoms between the myocardial and non-myocardial injury groups, except for fatigue and dyspnea (Table 2). Although no significant difference in pulse was found on admission, the incidence of tachycardia during hospitalization was significantly increased in patients undergoing myocardial injury.

**Table 2:** Comparison in demographic and clinical characteristics of COVID-19 patients with and without myocardial injury.

	**Total**	**Myocardial injury**	**Non-myocardial injury**	***P*-value^**a**^**
	**(*n* = 400)**	**(*n* = 46)**	**(*n* = 354)**	
Age (yrs)	49.0 (37.0–61.0)	52.5 (42.8–68.0)	49.0 (36.0–60.0)	0.046
Female	191 (47.8%)	20 (43.5%)	171 (48.3%)	0.538
Hypertension	62 (15.5%)	12 (26.1%)	51 (14.1%)	0.035
Diabetes	37 (9.3%)	8 (17.4%)	29 (8.2%)	0.056
Hyperlipidemia	11 (2.8%)	4 (8.7%)	7 (2.0%)	0.028
Liver Disease	7 (1.8%)	2 (4.3%)	5 (1.4%)	0.187
Kidney disease	5 (1.3%)	3 (6.5%)	2 (0.6%)	0.012
**Signs and symptoms**				
Fever	334 (83.5%)	40 (87.0%)	294 (83.1%)	0.502
Cough	295 (73.8%)	33 (71.7%)	262 (74.0%)	0.724
Fatigue	91 (22.8%)	16 (34.8%)	75 (21.2%)	0.039
Abdominal discomfort/ diarrhea/vomiting	43 (10.8%)	6 (13.0%)	37 (10.5%)	0.612
Sore throat	35 (8.8%)	7 (15.2%)	28 (7.9%)	0.102
Weight (Kg)	65.0 (57.0–72.0)	65.0 (57.0–75.0)	65.0 (57.0–71.8)	0.889
Respiratory rate >20 breaths/min	162 (40.5%)	19 (42.2%)	143 (40.5%)	0.826
Pulse rate, median (bpm)	83.6 ± 12.9	86.3 ± 10.6	83.3 ± 13.1	0.171
Peak pulse rate, (bpm)	97.3 ± 11.9	103.2 ± 14.0	96.8 ± 11.6	0.012

a *Significant difference (p < 0.05) was determined between the myocardial and non-myocardial injury groups*.

### Laboratory and Electrocardiographic Findings Showing Cardiac Dysfunction

COVID-19 patients with myocardial injury showed markedly increased levels of interleukin-6 [6.5 (5.2–17.9) vs. 2.3 (1.5–6.3) pg/mL; *P* < 0.001]. However, there was no significant difference on the levels of CRP, an acute phase protein known to arise during inflammation, between the cardiac injury and non-injury groups. We observed that patients with elevated TnI/T also had increased blood levels of other types of biomarkers for cardiac injury and dysfunction [e.g., lactate dehydrogenase, creatine kinase, and *N*-terminal pro-B-type natriuretic peptide (NT-proBNP)]. Compared with non-myocardial injury patients, the abnormality of lipid metabolites in peripheral blood occurred at a higher frequency in myocardial injury patients, with raising levels of total cholesterol (4.7 ± 1.1 vs. 4.0 ± 2.5 mmol/L; *P* = 0.029), low-density lipoprotein (2.8 ± 1.0 vs. 2.2 ± 0.7 mmol/L; *P* = 0.001), and triglycerides [2.7 (1.5–4.1) vs. 1.1 (0.9–1.9) mmol/L; *P* < 0.001] (Table 3).

**Table 3:** Laboratory and electrocardiographic findings of COVID-19 patients with or without myocardial injury.

	**Total**	**Myocardial injury**	**Non-myocardial injury**	***P*-value^**a**^**
	**(*n* = 400)**	**(*n* = 46)**	**(*n* = 354)**	
**Laboratory findings**				
White blood cells, mean, × 10^9^/L	5.0 (4.0–5.8)	5.3 (3.8–6.7)	5.0 (4.0–5.8)	0.455
Neutrophils, mean, × 10^9^/L	3.2 (2.2–4.2)	3.3 (2.3–5.2)	3.2 (2.2–4.2)	0.567
Lymphocytes, mean, × 10^9^/L	1.0 (0.9–1.5)	1.0 (0.8–1.3)	1.0 (0.9–1.5)	0.058
Platelets, median, × 10^9^/L (*n* = 388)	173.5 ± 59.3	166.8 ± 61.4	174.4 ± 59.1	0.420
Alanine aminotransferase, U/L (*n* = 392)	24.0 (15.0–39.5)	20.0 (14.8–28.3)	24.0 (15.0–41.0)	0.130
Aspartate aminotransferase, U/L (*n* = 392)	26.0 (19.0–34.0)	27.5 (21.0–34.2)	26.0 (19.0–34.0)	0.259
Creatinine, μmol/L (*n* = 391)	57.6 (46.9–71.3)	66.4 (51.8–76.4)	56.9(46.1–70.0)	0.007
Creatine kinase, U/L (*n* = 391)	61.0 (41.0–100.0)	84.0 (54.6–150.8)	60.0 (39.5–92.9)	0.002
Lactate dehydrogenase, U/L (*n* = 391)	191.0 (154.0–263.0)	227.0 (167.5.0–311.5)	188.0 (152.5–256.0)	0.015
Interleukin-6, pg/mL (*n* = 103)	5.2 (1.5–7.2)	6.5 (5.2–17.9)	2.3 (1.5–6.3)	<0.001
C-reactive protein, mg/L (*n* = 393)	18.5 (4.6–38.8)	20.7 (5.8–43.3)	18.4 (4.1–37.8)	0.709
NT-Pro-BNP, pg/mL (*n* = 80)	68.0 (27.3–330.5)	663.6 (103.8–2450.5)	51 (24.2–179.8)	<0.001
Total cholesterol, mmol/L (*n* = 211)	4.1 ± 2.4	4.7 ± 1.1	4.0 ± 2.5	0.029
Low-density lipoprotein, mmol/L (*n* = 210)	2.2 ± 0.8	2.8 ± 1.0	2.2 ± 0.7	0.001
Triglycerides, mmol/L (*n* = 211)	1.1 (0.9–2.2)	2.7 (1.5–4.1)	1.1 (0.9–1.9)	<0.001
Arterial pH, (*n* = 80)	7.46 ± 0.05	7.45 ± 0.05	7.46 ± 0.05	0.322
SaPO_2_ <95%	28/167 (16.8%)	7/18(38.9%)	21/149(14.1%)	0.015
**Electrocardiographic findings**				
ST-T change	20/106 (18.9%)	1/16(6.3%)	19/90(21.1%)	0.296
Left bundle branch block	3/106 (2.8%)	0/16 (0.0%)	3/90(3.3%)	>0.999

a *Significant difference (p < 0.05) was determined between the myocardial and non-myocardial injury groups*.

Of 106 patients with the electrocardiogram records, 20 (18.9%) patients developed ST-T changes. However, the distribution was not significantly different between patients with and without elevated cTnI/T levels.

COVID-19 patients with myocardial injury showed no change in the pH values of arterial blood while having a higher prevalence of hypoxia (SaPO2 <95%) than those without cardiac injury (Table 3), implying increased severity of COVID-19 injury toward the respiratory system in patients with myocardial injury.

### Chest CT Scan Assessment of EATV Predicating Myocardial Injury

**Figure 2:**
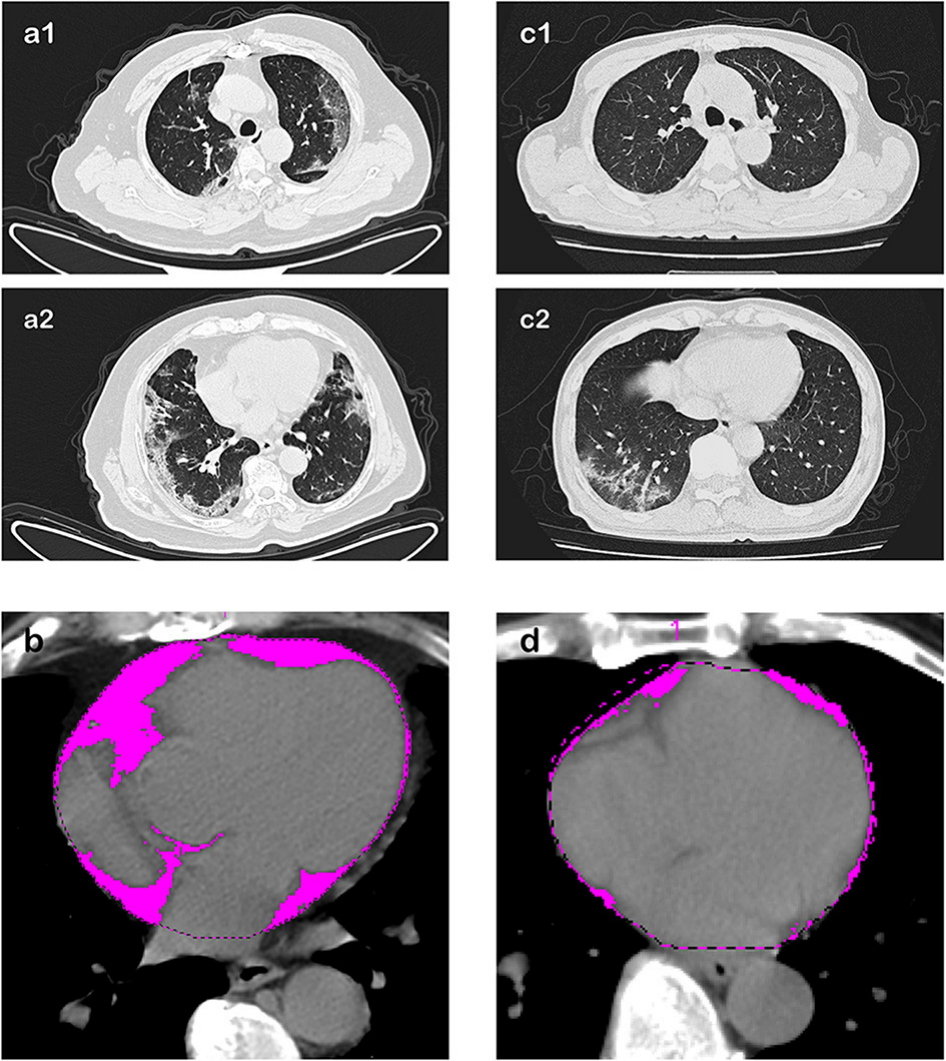
Chest CT scan images and EATV assessment in COVID-19 patients with and without myocardial injury. Transverse chest CT images at two different positions were collected during the first 24 h of hospital admission. Epicardial adipose tissue volume was assessed and calculated in the pink area. Case A, in the age range of 60–70 years, who presented myocardial injury during hospitalization, representative chest CT scan image at two different pulmonary and cardiac positions: **(a1)** Upper position of heart and lung, and **(a2)** middle-lower position of heart and lung), showing subpleural strip-like ground-glass opacities and patchy consolidation scattering in the left upper lobe, right middle lobe, and bilateral lower lobes, the typical imaging findings of lung involvement in COVID-19. **(b)** The epicardial adipose tissue volume was 221 cm3, presented as the pink area. Case B, also in the age range of 60–70 years but did not present myocardial injury during hospitalization. **(c1,c2)** Representative CT scan images at the lung window showing similar images of lung injury characteristic of COVID-19: subpleural irregular patchy and grid-like hyperdense shallows with blurred edges in the right lower lobe. **(d)** The epicardial adipose tissue volume or EATV was 86.5 cm3 in this case. The volume of hypodense tissue surrounding the myocardium appearing much smaller than that shown in case A.

The chest CT scan performed on admission showed that EATV in patients with myocardial injury was significantly larger than the non- injury patients [139.1 (83.8–195.9) vs. 92.6 (76.2–134.4) cm^2^; *P* = 0.036]. Figure 2 illustrates chest CT images in COVID-19 cases with and without myocardial injury. Using the receiver operating characteristic (ROC) curve analysis, we found that a cut-off value of 137.1 cm^2^ in EATV had predicted the occurrence of myocardial injury at 55% sensitivity, 77% specificity, and the area under the curve of 0.642. The positive likelihood ratio is 0.193, while the negative likelihood ratio is 0.046. Patients with EATV over 137.1 cm^2^ on admission were more commonly diagnosed with myocardial injury than those not [11/68 (16.2%) vs. 9/204 (4.4%); *P* = 0.001]. In the univariable logistic analysis, odds of myocardial injury were greater in patients with EATV on admission over 137.1 cm^2^. Age, and pre-existing health conditions, such as diabetes, hyperlipidemia, and coronary heart disease, were also significantly associated with myocardial injury. In a multivariable logistic regression model which included 265 patients with necessary data (20 with myocardial injury and 245 without myocardial injury), we found that EATV on admission over 137.1 cm^2^ was associated with the higher incidence of myocardial injury [adjusted odds ratio (OR) 3.058, (95%CI, 1.032–9.063); *P* = 0.044], after adjusting the influence of age, body weight, the history of coronary heart disease and hyperlipidemia. Age and the history of hyperlipidemia also remained significant in this model (Table 4).

**Table 4:** Predictors for the incidence of myocardial injury (*n* = 265).

	**Univariable OR (95% CI)**	***P-*value^**a**^**	**Multivariable OR (95% CI)**	***P*-value^**b**^**
Age, 10 years	1.225 (1.004–1.495)	0.045	1.602 (1.035–2.477)	0.034
Male sex (vs female)	1.215 (0.654–2.256)	0.538	–	–
Hyperlipidemia (*vs* not present)	4.721 (1.326–16.803)	0.017	5.247 (1.122–24.551)	0.035
Coronary heart disease (*vs* not present)	8.000 (1.099–58.224)	0.040	8.273 (0.742–92.187)	0.086
Epicardial adipose tissue volume on admission >137.1 cm^2^ (vs. not present)	4.181 (1.651–10.588)	0.003	3.058 (1.032–9.063)	0.044
Weight	0.997 (0.972–1.023)	0.814	1.021 (0.973–1.072)	0.402

*OR, odd ratio; CI, confidence intervals*.

a *Significant difference (p < 0.05) was determined using univariable logistic regression model*.

b *Significant difference (p < 0.05) was determined using multivariable logistic regression model*.

### Therapeutic Approaches and Outcomes in COVID-19 Patients With and Without Myocardial Injury

Almost all the enrolled patients received various antiviral treatments. No differences in therapeutics were found between the myocardial injury and non-myocardial injury groups, except for the usage of corticosteroids [17/46 (37.0%) vs. 83/354 (23.4%); *P* = 0.047] (Table 5). In myocardial injury patients, corticosteroid therapies had markedly decreased the blood levels of IL-6 [6.0 (4.9–7.6) vs. 15.4 (5.8–34.9) pg/mL; *P* = 0.03] as well as the incidence of in-hospital adverse events [1/17 (5.9%) vs. 11/29 (37.9%); *P* = 0.034].

**Table 5:** Therapeutics received and outcomes of COVID-19 patients with or without myocardial injury.

	**Total (*n* = 400)**	**Myocardial injury (*n* = 46)**	**Non-myocardial injury (*n* = 354)**	***P*-value^**a**^**
**Treatment**				
Oxygen therapy	199 (49.8%)	24 (52.2%)	175 (49.7%)	0.754
Invasive mechanic ventilation	5 (1.3%)	1 (2.2%)	4 (1.1%)	0.459
Non-invasive mechanic ventilation	25 (6.3%)	2/46 (4.3%)	23 (6.5%)	0.459
Lopinavir/ritonavir	236 (59.0%)	29 (63.0%)	207 (58.5%)	0.553
Arbidol	160 (40.0%)	22 (47.8%)	138 (39.0%)	0.266
Oseltamivir	94 (23.5%)	14 (30.4%)	80 (22.6%)	0.238
Antibiotics	282 (70.6%)	31 (67.4%)	251 (71.1%)	0.608
Corticosteroids	100 (25.0%)	17 (37.0%)	83 (23.4%)	0.047
**Outcomes**				
ICU admission	40 (10.0%)	11 (23.9%)	29 (8.2%)	0.003
Death	8 (2.0%)	5 (10.9%)	3 (0.8%)	0.001

*ICU, intensive care unit*.

a *Significant difference (p < 0.05) was determined between the myocardial and non-myocardial injury groups*.

In-hospital adverse events (admission to an ICU, invasive mechanical ventilation, or death) occurred in 47 patients (11.8%), including 40 (10.0%) of whom were admitted to ICU, 5 (1.3%) underwent invasive mechanical ventilation, and 8 (2.0%) died (Table 5). Compared with those without myocardial injury, myocardial injury patients underwent more in-hospital adverse events [12/46 (26.1%) vs. 35/354 (9.9%); *P* = 0.001], while the incidence of death and ICU admission were higher too.

In the multivariable logistic regression model including all 400 patients (46 patients with myocardial injury and 354 without myocardial injury), the myocardial injury was independently associated with the risk of in-hospital adverse events [adjusted OR 2.607 (95%CI: 1.166–5.830); *P* = 0.020] after adjusting for age, sex, history of hypertension, diabetes and coronary heart disease. Age also remained significant in this model, indicating that it also contributes to in-hospital adverse events (Supplementary Table 2). This association remained stable in patients with EATV value (*n* = 272) in subgroup analysis (Supplementary Table 3).

## Discussion

In the current cohort study we investigated and compared the clinical characteristics between COVID-19 patients with and without myocardial injury, who were admitted to six hospitals and regional medical centers outside of the epicenter of Wuhan. This group of patients demonstrated certain pathophysiological characteristics, to a certain degree, different from those hospitalized and treated in Wuhan or other epicenters of COVID-19 around the world. We observed that many of the patients had pre-existing health conditions and increased values of EATV on admission which might be predisposed to the pathogenesis of myocardial injury. The average age of patients with myocardial injury appears higher than those without myocardial injury, but the age gap only marginable, suggesting that in this cohort, pre-existing health conditions, rather than age, might serve as the major risk factors for the development of myocardial injury. Pre-existing cardiovascular and metabolic comorbidities were more commonly observed in COVID-19 patients with myocardial injury, along with abnormal levels of metabolic indicators, indicating COVID-19 patients with underlying cardiovascular conditions, especially abnormal lipid metabolism, are exposed to an increased risk for myocardial injury. Myocardial injury serves as a contributor to the severity and mortality of COVID-19, with reported hazard ratio ranging from 2.1 to 8.9 (9, 16, 17), and odds ratio from 6.6 to 26.9 (18–20) in different studies. Our logistic regression analysis also suggests that myocardial injury is an independent adverse event, which precipitates poor prognosis. Thus, it is of great importance to timely detect and treat patients with a high risk of myocardial injury and to offer a special care to avoid relevant adverse events.

Patients with the deadly contagious disease COVID-19 often receive medical attention in ICU or emergency room. Upon admission, less likely, they will have a comprehensive imaging assessment of cardiac complications, including echocardiography and CMR. Moreover, echocardiographic findings in patients with myocardial injury are mostly non-specific (9). Slight injury may not lead to functional or structural changes, and often it is undetectable by echocardiography and cardiac magnetic reasoning imaging. Only 20% of COVID-19 patients with myocardial injury showed abnormality on echocardiogram, left others with normal performance (21). CMR is reportedly helpful in revealing the cardiac involvement of COVID-19 in recovered patients, but its predicting value in COVID-19 patients is doubtful (22).

In the current cohort study, we explored the feasibility of using cardiac images from routine chest CT scan as a potential index of myocardial injury. Our findings demonstrate the correlation between EATV on admission and the occurrence of myocardial injury. First, the mean value of EATV is significantly larger in COVID-19 patients with myocardial injury than those without myocardial injury. Second, 137.1 cm^2^ is the optimal cut-off point of EATV for predicting in-hospital myocardial injury on ROC analysis. Third, EATV over 137.1 cm^2^ is the strong independent indicator for myocardial injury in general COVID-19 patients, with a valuable negative predictive value.

For the diagnosis and assessment of pneumonia, the predominant manifestation of COVID-19, patients are routinely examined by chest CT scan. Strictly speaking, EATV is a measurement of not mere fat tissue expansion but also peri- or epicardiac soft tissue (perhaps consisting of both fat and inflammatory connective tissues) enlargement with inflammatory responses (10, 23). It is exquisitely sensitive to the adjacent inflammatory states associated with coronary atherosclerotic plaque, atrial fibrillation, and systemic inflammatory disorders (24).

To date, the precise mechanisms that cause myocardial injury in COVID-19 patients are not entirely understood. The cytokine storm (i.e., excessive and uncontrollable cytokine production in response to SARS-CoV-2 infection, may be one of the main contributors to the pathogenic injury of myocardium). There have been plenty of studies indicating that serum levels of cytokines are significantly increased in COVID-19 patients (3, 4). Moreover, cytokine levels were associated with disease mortality and the incidence of myocardial injury (2, 25, 26), indicating the contributing role of cytokine storm in COVID-19 associated myocardial injury. In our population, compared with patients without myocardial injury, IL-6 levels were significantly higher in myocardial injury patients, implying the possible pathogenic role of the cytokine storm in the development of myocardial injury. CRP levels were increased too, but statistically no significance was found between the groups of COVID-19 patients. Myocardial injury patients treated with corticosteroids had markedly decreased levels of IL-6. This observation may partially explain the improved outcome in myocardial injury patients treated with the steroids.

Epicardial fat may represent a transducer that mediates the detrimental impacts of systemic inflammation on the adjacent myocardium (27). We observed the significantly enlarged EATV in COVID-19 patients with myocardial injury, which may be due to inflammatory cell infiltration and temporary edema related to systemic cytokine storm and pericarditis and micro-myocarditis.

Increased EATV has been shown in obese individuals with increased chest and abdominal obesity, a possible risk factor for myocardial injury. Abdominal obesity is proved to be the major risk factor for disease progression and mortality in COVID-19 patients, independent of obesity-related comorbidities (28, 29). So high body mass index (BMI) and waist-hip ratio indicate a high risk of hospitalization (30). As a reflection of total visceral fatness, EATV is associated with BMI and waist circumference (31, 32), so the strong association between high EATV and myocardial injury may reveal the possible contributing role of overall and abdominal obesity to the development of myocardial injury. In this study, we observed hyperlipidemia in COVID-19 patients with myocardial injury. The elevation of EATV values in COVID-19 patients may also reflect this pathological condition.

Taken together, observations from the current study clearly document that EATV enlargement may serve as a potentially important parameter or predictor for the development of myocardial injury. Although the exact mechanism behind the association of high EATV and in-hospital myocardial injury remains unclear, it is recommendable to employ the CT scan measurement of EATV as an early risk evaluation for myocardial injury in COVID-19, in combination with other imaging methods.

### Study Limitations

First, given the retrospective nature of this study, some parameters were not available in all the patients enrolled in the study. There were 128 enrolled patients who lacked the mediastinal window images, so the predicting value of EATV was analyzed based on data from the other 272 patients. Systemic bias might be introduced, though the baseline characteristics between patients with and without EATV values were roughly the same. Second, the inconsistency of troponin type between study centers deters us from clarifying the correlation between EATV and the severity of myocardial injury, which may offer a more comprehensive picture for EATV study in COVID-19 patients with cardiovascular complications. Third, myocardial injury was identified by a combination of biomarkers and clinical symptoms, primarily the abnormal levels of TnI/T during hospitalization. However, TnI/T levels could be affected by other determinants, such as the infection status, hypoxia, and renal insufficiency, which might lead to the false-positive diagnosis. On the other hand, false positive diagnosis might exist as some patients approaching to stable conditions might have a decreased likelihood of myocardial injury identification. This could cause a systematic bias when assessing the relationship between myocardial injury and disease severity. Fourth, echocardiographic data were not available in enrolled patients. A comprehensive assessment of the heart function using electrocardiography, imaging, and laboratory testing would help a deeper understanding of clinical profiles of myocardial injury.

Furthermore, we only account for weight in logistic regression, instead of other better indicator for obesity like BMI or waist-hip ratio. So as an early predictor for myocardial injury, EATV may not be independent of obesity. Whether simple anthropometric data is a predictor for myocardial injury will be explored in our further study. The role of abdominal obesity in myocardial injury development is also worthy of being investigated in the future, leveraging specific indicators like adiponectin. And finally, the cohort is relatively smaller and restricted to the Han Chinese COVID-19 patients. Thus, the conclusion should be further confirmed by large-scale prospective cohort studies in ethnically diverse cohorts.

## Conclusions

Myocardial injury is the major in-hospital adverse event that contributes to the mortality of COVID-19 patients. Pre-existing health conditions, inflammatory cytokine production, and augmented EATV on admission may serve as potentially independent risk factors for the development of myocardial injury in COVID-19 patients. EATV at less than the threshold 137.1 cm^2^ or so in a chest CT scan on admission may predict a better outcome for COVID-19 patients with increased risks of myocardial injury.

## Data Availability Statement

The raw data supporting the conclusions of this article will be made available by the authors, without undue reservation.

## Ethics Statement

The studies involving human participants were reviewed and approved by Ethics committees of Yichang Central People's Hospital, the First Affiliated Hospital of University of Science and Technology of China, Daye Chinese Medicine Hospital, Anqing Hospital, Baoding No.1 Central Hospital and Fifth Affiliated Hospital of Sun Yat-sen University. Written informed consent for participation was not provided by the participants' legal guardians/next of kin because: Written informed consent was waived due to the rapid emergence of this infectious disease, and data analysis were performed anonymously.

## Author Contributions

Z-YW, H-YQ, W-XY, and Y-JG designed the study. Z-YW, H-YQ, and Y-JG drafted the manuscript. RQ, JC, W-JW, HY, JX, HW, CW, and C-HG acquired and analyzed the data. Z-YW and YW contributed to the statistical analysis. JH, ML, CL, JY, H-MD, M-JL, K-WL, and H-FS made technical support. All authors contributed to the article and approved the submitted version.

## Conflict of Interest

The authors declare that the research was conducted in the absence of any commercial or financial relationships that could be construed as a potential conflict of interest.
