# Supplementary material for: Pre-existing Health Conditions and Epicardial Adipose Tissue Volume: Potential Risk Factors for Myocardial Injury in COVID-19 Patients
Source: Front Cardiovasc Med. 2021 Jan 11;7:585220. doi: 10.3389/fcvm.2020.585220 (PMC7829196; doi:10.3389/fcvm.2020.585220)
Supplement: Supplementary file 1 [file Data_Sheet_1.docx]

**Supplementary Table 1. Different reference ranges of TnI/T in 6 centers**

| **Hospital** | **Troponin reference** | **Testing** |
| --- | --- | --- |
| Yichang Central People’s Hospital | TnT :0-14pg/ml | Electrochemiluminescence, Roche Diagnostics |
| the First Affiliated Hospital of USTC | TnI : 0-0.3ng/ml | Chemiluminescence, Shenzhen New Industry Biomedical Engineering Co. |
| Daye Chinese Medicine Hospital (center 1) | TnI: 0-1ng/mL | Immunofluorescence, GeteinBiotech |
| Daye Chinese Medicine Hospital (center 2) | TnI : 0.3 ng/ml | Chemiluminescence, Shenzhen New Industry Biomedical Engineering Co. |
| Anqing Hospital | TnT : 0-14pg/ml | Electrochemiluminescence, Roche Diagnostics |
| Baoding No.1 Central Hospital | - | - |
| Fifth Affiliated Hospital of Sun Yat-sen University | TnI : 0-0.0229ng/mL | DELFIA, Radiometer |

Abbreviation: TnI/T = troponin I/T

**Supplementary Table 2. Risk factors associated with composite endpoints (n=400)**

|  | **Univariable OR (95% CI)** | **p value** | **Multivariable OR (95% CI)** | **p value** |
| --- | --- | --- | --- | --- |
| Age, 10 years | 1.667 (1.334-2.083) | <0.001 | 1.576 (1.242-1.999) | <0.001 |
| Male sex (vs female) | 0.958 (0.521-1.763) | 0.891 | - | - |
| Hypertension (*vs* not present) | 2.632 (1.315-5.269) | 0.006 | 1.441 (0.667-3.112) | 0.352 |
| Diabetes (*vs* not present) | 2.292 (0.979-5.364) | 0.056 | 1.266 (0.498-3.214) | 0.620 |
| Coronary heart disease (*vs* not present) | 2.536 (0.258-24.894) | 0.424 | 2.116 (0.158-28.353) | 0.571 |
| Myocardial injury (*vs* not present) | 3.217 (1.527-6.775) | 0.002 | 2.607 (1.166-5.830) | 0.020 |

**Supplementary Table 3. Risk factors associated with composite endpoints in patietns with EATV value (n=272)**

|  | **Univariable OR (95% CI)** | **p value** | **Multivariable OR (95% CI)** | **p value** |
| --- | --- | --- | --- | --- |
| Age, 10 years | 1.762 (1.336-2.323) | <0.001 | 1.593 (1.184-2.144) | 0.002 |
| Male sex (vs female) | 1.017 (0.504-2.051) | 0.962 | - | - |
| Hypertension (*vs* not present) | 2.614 (1.178-5.799) | 0.018 | 1.388 (0.564-3.416) | 0.475 |
| Diabetes (*vs* not present) | 3.460 (1.377-8.692) | 0.008 | 1.969 (0.695-5.582) | 0.202 |
| Coronary heart disease (*vs* not present) | 2.219 (0.225-21.933) | 0.495 | 4.669 (0.273-79.873) | 0.287 |
| Myocardial injury (*vs* not present) | 6.818 (2.592-17.935) | <0.001 | 4.211 (1.445-12.267) | 0.008 |

Abbreviation:OR=odd ratio; CI= confidence interval
